# Mobile Telemedicine for Buprenorphine Treatment in Rural Populations With Opioid Use Disorder

**DOI:** 10.1001/jamanetworkopen.2021.18487

**Published:** 2021-08-27

**Authors:** Eric Weintraub, Chamindi Seneviratne, Jessica Anane, Kelly Coble, Jessica Magidson, Sarah Kattakuzhy, Aaron Greenblatt, Christopher Welsh, Alexander Pappas, Terri L. Ross, Annabelle M. Belcher

**Affiliations:** 1 Division of Addiction Research and Treatment, Department of Psychiatry, University of Maryland School of Medicine, Baltimore; 2 Department of Psychology, University of Maryland, College Park; 3 Division of Critical Care and Research, Institute of Human Virology, Division of Infectious Disease, University of Maryland School of Medicine, Baltimore; 4 Now with Venice Family Clinic, Venice, California; 5 Caroline County Health Department, Denton, Maryland

## Abstract

**Importance:**

The demand for medications for opioid use disorder (MOUD) in rural US counties far outweighs their availability. Novel approaches to extend treatment capacity include telemedicine (TM) and mobile treatment on demand; however, their combined use has not been reported or evaluated.

**Objective:**

To evaluate the use of a TM mobile treatment unit (TM-MTU) to improve access to MOUD for individuals living in an underserved rural area.

**Design, Setting, and Participants:**

This quality improvement study evaluated data collected from adult outpatients with a diagnosis of OUD enrolled in the TM-MTU initiative from February 2019 (program inception) to June 2020. Program staff traveled to rural areas in a modified recreational vehicle equipped with medical, videoconferencing, and data collection devices. Patients were virtually connected with physicians based more than 70 miles (112 km) away. Data analysis was performed from June to October 2020.

**Intervention:**

Patients received buprenorphine prescriptions after initial teleconsultation and follow-up visits from a study physician specialized in addiction psychiatry and medicine.

**Main Outcomes and Measures:**

The primary outcome was 3-month treatment retention, and the secondary outcome was opioid-positive urine screens. Exploratory outcomes included use of other drugs and patients’ travel distance to treatment.

**Results:**

A total of 118 patients were enrolled in treatment, of whom 94 were seen for follow-up treatment predominantly (at least 2 of 3 visits [>50%]) on the TM-MTU; only those 94 patients’ data are considered in all analyses. The mean (SD) age of patients was 36.53 (9.78) years, 59 (62.77%) were men, 71 (75.53%) identified as White, and 90 (95.74%) were of non-Hispanic ethnicity. Fifty-five patients (58.51%) were retained in treatment by 3 months (90 days) after baseline. Opioid use was reduced by 32.84% at 3 months, compared with baseline, and was negatively associated with treatment duration (*F* = 12.69; *P* = .001). In addition, compared with the nearest brick-and-mortar treatment location, TM-MTU treatment was a mean of 6.52 miles (range, 0.10-58.70 miles) (10.43 km; range, 0.16-93.92 km) and a mean of 10 minutes (range, 1-49 minutes) closer for patients.

**Conclusions and Relevance:**

These data demonstrate the feasibility of combining TM with mobile treatment, with outcomes (retention and opioid use) similar to those obtained from office-based TM MOUD programs. By implementing a traveling virtual platform, this clinical paradigm not only helps fill the void of rural MOUD practitioners but also facilitates access to underserved populations who are less likely to reach traditional medical settings, with critical relevance in the context of the COVID-19 pandemic.

## Key Points

**Question:**

Can medications to treat opioid use disorder be effectively provided to individuals living in underserved rural areas via telemedicine in a mobile treatment unit?

**Findings:**

In this quality improvement study, comparable to office-based telemedicine programs, 58.51% of patients treated in a mobile telemedicine treatment unit remained in treatment at 90 days. Longer retention was significantly associated with reduced opioid use.

**Meaning:**

These findings suggest that the combination of telemedicine and mobile services is a unique approach to extend access to medications for opioid use disorder to rural areas and is especially relevant in a postpandemic climate; this model demonstrates feasibility and lays the groundwork for adoption in rural populations.

## Teaser

This quality improvement study evaluates the use of a telemedicine mobile treatment unit to improve access to medications for opioid use disorder for individuals living in an underserved rural area.

## Introduction

Rural regions of the US have been disproportionately affected by the misuse of prescription and illicit opioids, with increased annual rates of per capita overdose deaths since 1999.^1,2^ Opioid agonist medications (medications for opioid use disorder [MOUD]) approved for the treatment of opioid use disorder (OUD) include methadone and buprenorphine, both of which have demonstrated efficacy in decreasing opioid use and risk of overdose and death.^3,4^ However, treatment programs are in short supply in rural areas^5^; more than 50% of rural US counties are without a single buprenorphine-waivered practitioner.^6,7,8^ Moreover, it is estimated that 10% of the US population resides more than 10 miles (16 km) from the nearest buprenorphine practitioner, with 2.65 million individuals located more than 30 miles (48 km) away.^9^ This dearth of resources has rendered these communities poorly equipped to meet the increasing demand for MOUD.

Telemedicine has been identified as an evidence-based strategy to improve access to MOUD in hard-to-reach settings and populations.^10,11,12,13^ To date, our clinicians have treated more than 500 patients with the combination of telemedicine (TM) via videoconferencing and remote buprenorphine prescription in underserved rural counties. Patients enrolled in these TM MOUD clinical services receive the expert care of addiction medicine and/or psychiatry physicians via doctor-on-a-screen encounters that are conducted in treatment facilities located in the rural communities. Published data^12,13^ suggest that TM MOUD produces clinical outcomes (specifically, retention and decreased illicit substance use) that are comparable to those of in-person treatment.

Despite the success of office-based TM MOUD programs, there remains a persistent gap in awareness of these services. Perhaps most critical is travel, a barrier further heightened in rural areas without adequate public transit infrastructure. In the outlying Eastern Shore regions of Maryland that are the focus of our project, public transit systems are simply nonexistent, making it challenging for individuals to arrive to office-based programs for scheduled TM encounters. In a recent assessment of 507 Eastern Shore Maryland residents, transportation was listed as the second highest challenge in the lived environment.^14^ Furthermore, with minimal entry points into the community, this region is not well-served by infrastructure to access or deliver health care. For example, there are only 2 highways spanning the entire Eastern Shore, with some communities accessible only by boat. As a poignant example of the burden caused by lack of transportation, 1 patient participating in office-based TM was relegated to using farm equipment (a tractor) to arrive for scheduled treatment appointments. This persistent issue drove our team to look for creative ways in which to further extend the outreach of our TM capability and to decrease the burden associated with locating transportation for substance use treatment.

Here we present a novel strategy to further extend access to TM MOUD to rural communities, by bringing care via a TM mobile treatment unit (TM-MTU). Although mobile MOUD paradigms have been described before,^15,16^ our approach integrates TM, thus obviating the need for on-site buprenorphine-waivered practitioners. This analysis seeks to evaluate the initial implementation of this initiative, including treatment and adherence outcomes data on the first cohort of individuals treated in the TM-MTU program.

## Methods

### Study Population and Design

This quality improvement study was performed from June to October 2020. We report data obtained from all patients who entered treatment with TM-based medications for OUD onboard our MTU. Deidentified data are stored on a database maintained at the University of Maryland School of Medicine. Data in this report were collected as part of an exempt-determined study protocol, provided following 45 CFR 46.101b guidelines by the University of Maryland Human Research Protection Office; thus, the need for informed consent was waived. Results are reported following the Standards for Quality Improvement Reporting Excellence (SQUIRE) reporting guidelines.^17^

### Intervention: The TM-MTU

**Figure 1.**
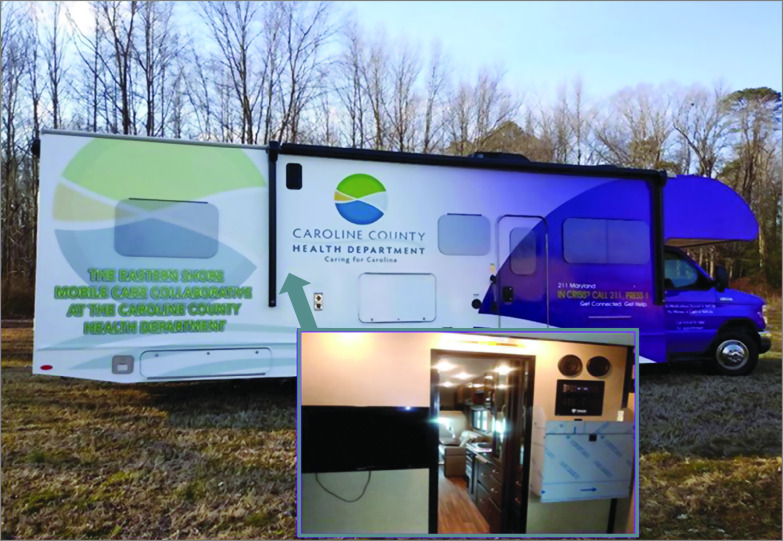
The Telemedicine Mobile Treatment Unit (TM-MTU) The inset shows the interior of the TM-MTU, which allows space for a waiting area, a kitchen with a refrigerator, a bathroom that can be used for observed urine collection, and a private meeting room for video encounters (TM appointments) between patients and physicians from the Division of Addiction Research and Treatment. All interactive video conferencing sessions are conducted point-to-point using Health Insurance Portability and Accountability Act–compliant Advanced Encryption Standard algorithm via internet protocol connections. The mobile unit backup power supply uses a Tripp Lite Medical-Grade kit, which includes 2 modules: a power supply module and a battery module. When the 2 modules are connected (and attached to a remote user interface), they provide AC power for a variety of equipment on the MTU.

The Caroline County TM-MTU initiative is an ongoing collaboration between the University of Maryland School of Medicine Division of Addiction Research and Treatment in Baltimore, Maryland, and the Caroline County Health Department (CCHD). A recreational vehicle was modified to serve as the TM-MTU (Figure 1). Patients are enrolled into the treatment program through various advertising efforts in the local community and referrals from emergency departments and local jails. Patient scheduling is usually done before the TM-MTU visits, or walk-in patients can be seen on the same day.

The TM-MTU is staffed with 3 trained individuals: a nurse, a peer recovery specialist, and a substance use counselor. The nurse records vital signs, schedules patients for TM appointments, and conducts urine drug screens with point-of-care tests. The peer recovery specialist provides patient-centered recovery support services to promote the feasibility of the TM-MTU services and operations. The substance use counselor screens patients, provides individual counseling, administers intake and other questionnaires, and schedules patient TM MOUD encounters. All patients meet with both the counselor and peer recovery specialist, and all procedures adhere to formalized standard operating procedures developed for the program.

Since the TM-MTU became operational in February 2019 in Denton, Maryland (the seat of Caroline County), treatment services have expanded to the towns of Greensboro, Federalsburg, and Ridgely to cover large parts of Caroline County by June 2020. At these locations, the TM-MTU is stationed in public areas (1 of 4 church parking lots located in each of the 4 towns) and accommodates up to 2 patients at a time. Each TM session for a new evaluation takes approximately 45 minutes, and follow-up sessions typically take 20 minutes. Once a TM session is completed, a buprenorphine or naloxone prescription is electronically prescribed to a local pharmacy, and patients pick up their own medications. The TM-MTU functions as a satellite entity of the CCHD and, thus, is governed by the same Drug Enforcement Administration registration, which permits prescribing of buprenorphine without an in-person examination. As with office-based services, TM-MTU clinical services are fully covered by Medicare, Medicaid, and private insurance programs.

The MTU on-site staff members coordinate with the Division of Addiction Research and Treatment program managers to schedule patient visits, as determined by the physician. Urine samples are sent out for toxicological analysis by enzyme-linked immunosorbent assay and liquid chromatography with tandem mass spectrometry, using a customized panel composed of 40 analytes (Ammons Labs; eTable 1 in the Supplement),^18^ the results of which are made available to the physicians.

Epic (Epic Systems Corp) and PatTrac (PatTrac) databases are used to enter patient notes and laboratory results and to schedule patient appointments. Data reported were collected as part of the retrospective chart review and stored on REDCap,^19^ a Health Insurance Portability and Accountability Act–compliant database for data entry and validation, storage, and retrieval.

### Outcome Measures

The primary outcome measure was retention in treatment, defined as continued engagement in treatment (with ≥1 follow-up visits), assessed at 0, 30, 60, and 90 days (3 months) after treatment intake. The secondary outcome measure was change in illicit opioid use during treatment as assessed by urine toxicology screens throughout the first 90 days of treatment. Exploratory outcome measures included urine toxicology results for other drug use, and patient travel time and distance to the preferred TM-MTU site location, as well as to the nearest brick-and-mortar treatment location. Determinations of race/ethnicity and sex were self-defined and supplied by participant self-report to clinical staff not involved in the research.

### Statistical Analysis

All statistical analyses were conducted using SAS/STAT statistical software version 9.4 (StataCorp). Because patients were given the option of follow-up treatment at the office-based CCHD, our patient population could have received varying levels of TM-MTU clinical services. To optimally discriminate cohort characteristics and report meaningful outcomes of treatment aboard the TM-MTU, we adopted a stringent approach to define who would be considered a TM-MTU patient, limiting the present analysis to those who were seen at least 50% of the time on the TM-MTU. Treatment retention rates were analyzed separately by calculating the percentages of patients who continued treatment at 7, 30, 60, 90, and 180 days after treatment initiation. To determine factors associated with treatment retention, we used a linear regression model that incorporated the following continuous variables: age and mean daily dose of buprenorphine prescribed throughout the duration of an individual’s treatment. Categorical variables in the model included buprenorphine use before treatment initiation (assessed by positive urine screens at baseline), sex, and race/ethnicity, because prior opioid treatment studies have reported that these variables may be associated with treatment outcomes.^20^

The analytes present in urine drug screens from TM-MTU patients were grouped into drug classes according to their chemical composition (eTable 1 in the Supplement). Any opioid, except buprenorphine or norbuprenorphine analyte, was considered an opioid-positive urine test. To assess changes in opioid use during treatment, percentages of treatment visits with opioid-positive urine screens were calculated for each patient separately and then correlated with the number of days retained in treatment. Because treatment visits were unevenly distributed among patients, the slope of regression curve was used to calculate percentages of subjects with opioid-free urine at days 7, 30, 60, and 90. Exploratory outcomes of patient travel times and miles are presented with descriptive statistics. Significance thresholds for all tests were set at *P* < .05 (2-tailed).

## Results

### Patient Population Characteristics

From the program’s inception date of February 1, 2019, to June 30, 2020, a total of 118 patients with an OUD were enrolled in the TM-MTU program. Of this population, 94 patients had treatment predominantly (at least 2 of 3 visits [>50%]) scheduled on the TM-MTU and were defined as the TM-MTU analysis cohort. There were no significant differences in demographic characteristics between the 94 participants (79.7%) in the TM-MTU cohort and the 24 participants (20.3%) in the non–TM-MTU cohort (eTable 2 in the Supplement). Of TM-MTU patients, the mean (SD) age was 36.53 (9.78) years, 59 (62.77%) identified as men, and 35 (37.23%) identified as women. Seventy-one individuals (75.53%) identified as White, 15 (15.96%) identified as Black or African American, 2 (2.13%) identified as multiracial, and 7 (6.38%) identified as other or unknown. Ninety individuals (95.74%) were of non-Hispanic ethnicity. Seventy-three of 78 patients (93.59%) tested positive for any opioid in baseline urine toxicology screens. The mean (SD) daily dose of buprenorphine prescribed was 15.24 (5.24) mg. Full patient baseline characteristics are presented in Table 1.

**Table 1.** Patient Characteristics

Characteristic	Patients, No. (%) (N = 94)
Age, mean (SD), y	36.53 (9.78)
Sex	
Male	59 (62.77)
Female	35 (37.23)
Race	
White	71 (75.53)
Black or African American	15 (15.96)
Multiracial	2 (2.13)
Unknown or other^a^	7 (6.38)
Ethnicity	
Hispanic	3 (3.19)
Non-Hispanic	90 (95.74)
Unknown	1 (1.06)
Patients with positive urine screens at baseline	
Alcohol	14 (17.95)
Amphetamines	15 (19.23)
Barbiturates	0
Benzodiazepines	6 (7.69)
Nonbenzodiazepine sedative	0
Cannabinoids	37 (47.44)
Cocaine	10 (12.82)
NMDA receptor antagonists	1 (1.28)
Opioids (including buprenorphine or norbuprenorphine)^b^	73 (93.59)
Opioids (excluding buprenorphine or norbuprenorphine)	45 (57.69)
Buprenorphine dose, mg	
Initial daily dose, mean (SD)	11.09 (4.84)
Daily dose across treatment, mean (SD)	15.24 (5.24)
Maximum daily prescribed dose	36.00
Minimum daily prescribed dose	4.00

Abbreviation: NMDA, N-methyl-D-aspartic acid.

^a^ The category of unknown or other did not include specific races or ethnicities.

^b^ Includes prescription and nonprescription opioids.

### Primary Outcome: Treatment Retention

**Figure 2.**
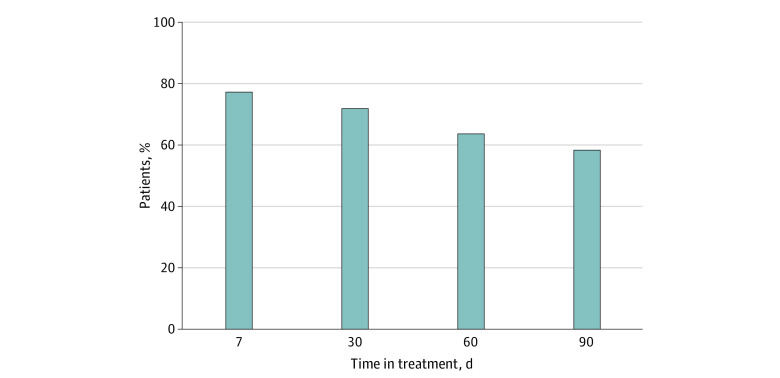
Primary Outcome Results: Telemedicine Mobile Treatment Unit Treatment Retention Each bar represents the percentage of 94 total patients retained at 5 different time points across treatment: 77.66% (73 patients) at 7 days, 72.34% (68 patients) at 30 days, 63.83% (60 patients) at 60 days, and 58.51% (55 patients) at 90 days after intake into treatment.

All 94 patients met the *Diagnostic and Statistical Manual of Mental Disorders* (Fifth Edition) criteria for OUD. From February 2019 to July 2020, patients were engaged in continuous treatment for a mean of 112.24 (74.06) days. Retention rates were 77.66% (73 patients) at 7 days, 72.34% (68 patients) at 30 days, 63.83% (60 patients) at 60 days, and 58.51% (55 patients) at 90 days from enrollment (Figure 2). These rates were not statistically significantly different from those in the enrollment population of 118 patients including TM-MTU and non–TM-MTU cohorts, with treatment retention rates of 82.20% (97 patients) at 7 days, 75.42% (89 patients) at 30 days, 66.95% (79 patients) at 60 days, and 58.47% (69 patients) at 90 days.

Within the group that returned for at least 1 follow-up visit, buprenorphine use at enrollment (indicated with a buprenorphine-positive urine analysis on the date of intake) was significantly positively associated with retention (*F* = 13.86; *P* < .001). In addition, mean prescribed buprenorphine dose was positively and significantly associated with longer treatment retention (*F* = 8.04; *P* = .006). Other factors, including sex, age, and race, did not show significant associations with retention (Table 2).

**Table 2.** Variables Associated With Treatment Retention^a^

Variable	Mean square	*F* value	*df*	*P* value
Overall model	13 185.21	1.57	7	.005
Prior buprenorphine use (as assessed by buprenorphine-positive urine analysis at enrollment)	56 868.22	13.86	1	<.001
Prescribed mean daily dose of buprenorphine	32 712.95	8.04	1	.006
Age	2662.44	0.30	1	.58
Sex	371.60	0.21	1	.65
Race/ethnicity	1025.45	0.09	3	.97

^a^ For the overall model, *R*^2^ = 0.25 and coefficient of covariance = 58.03.

### Secondary and Exploratory Outcome Measures: Changes in Drug Use, Travel Time, and Travel Miles

**Figure 3.**
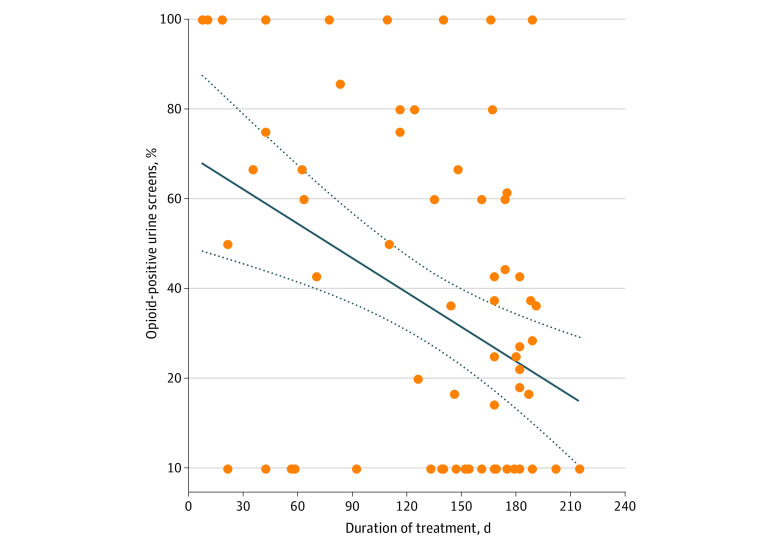
Secondary Outcome Results: Opioid Use During Telemedicine Mobile Treatment Unit Treatment Each dot represents the percentage of opioid-positive urine screens per individual patient averaged across their entire treatment period. Any opioid except buprenorphine or norbuprenorphine analyte was considered an opioid positive urine test. As calculated by using the slope for the regression line, the estimated percentages of opioid negative urine screens were 45.07% at 0 days, 56.72% at 7 days, 62.59% at 30 days, 70.25% at 60 days, and 77.91% at 90 days from enrollment. The solid line represents the regression line; dashed lines represent the 95% CI for the regression line.

As shown in Figure 3, opioid use was reduced by 32.84% at 3 months, compared with baseline, and was significantly negatively correlated with duration of treatment (*F* = 12.69; *P* = .001). On the basis of urine toxicology screens at each treatment visit, 62 MTU patients (65%) used at least 1 type of nonopioid drug during treatment (eTable 2 in the Supplement). The mean use was 2 types of nonopioid drugs, with a maximum of 5 types. Compared with their nearest MOUD (brick-and-mortar) treatment clinic, treatment visits on the MTU saved patients a mean of 6.52 travel miles (range, 0.10-58.70 travel miles) (10.43 km; range, 0.16-93.92 km) and 10 minutes (range, 1-49 minutes) of driving distance.

## Discussion

To our knowledge, the TM-MTU initiative is the first of its kind in the US, integrating both mobile and telehealth services to bring much needed treatment capacity to patients with OUD in underserved, difficult to access, rural communities. Data from the initial TM-MTU cohort demonstrate that, at 3 months, approximately 60% of patients remained in continuous treatment and reduced their opioid use by nearly one-third compared with baseline. Our results are comparable to 3-month treatment retention and opioid use reduction rates that we reported previously from brick-and-mortar office-based TM programs in similar rural communities.^12,13^ In addition, these positive outcomes are achieved at a considerable convenience to our patients, as evidenced by the afforded time and distance savings.

There were several noteworthy observations of TM-MTU retention rates. Of the 118 patients who initially sought treatment aboard the TM-MTU, approximately 20% (24 patients) were not seen after their initial treatment visit. Similar to reported retention rates of substance use treatment programs, most patient dropout occurred during the first 15 days of treatment.^21,22,23^ Although systematic identification of the reasons for early departures from treatment in our population would require assessments beyond those that we report here, continuing studies of patients in TM-MTU treatment who consent to an optional research component will assist in identifying some of those factors.

We found 2 factors in our patient population that were associated with TM-MTU treatment retention: pretreatment buprenorphine use and higher mean daily doses of buprenorphine. Several office-based studies^24,25^ have reported that a history of using nonprescribed buprenorphine may increase the likelihood of entering treatment and is associated with improved treatment retention. In addition, our finding that patients maintained on higher doses of buprenorphine are more treatment adherent is consistent with published literature.^26,27^ At less than 16 mg, however, the mean daily dose of buprenorphine prescribed to our patients is on the low end of buprenorphine prescribing doses. This could represent an area of clinical improvement and future research, to institute both a systematic assessment of patient satisfaction with dosage and a retention-enhancement protocol using higher dosages.

A notable strength of the TM-MTU program is its utility as a bridging service to support and enhance the services of office-based TM for MOUD, either when it is not accessible, or upon patients’ request. This allows patients who miss appointments or need more immediate clinical interventions to be examined expeditiously. The flexibility of this bridging service has been especially beneficial during the COVID-19 pandemic, when the CCHD clinic was closed. Unlike many treatment programs that were incapable of maintaining a sustained census through the pandemic, patients have continued to be seen on the TM-MTU while adhering to Centers for Disease Control and Prevention safety guidelines. In fact, at the time of this report, the TM-MTU program functions as the sole TM MOUD-providing entity in Caroline County and accommodates all patients enrolled in the various OUD treatment programs that are facilitated by the CCHD. Although several Food and Drug Administration–approved COVID-19 vaccines are now available, the possibility exists that it may take months to years to achieve the vaccination coverage necessary for herd immunity.^28^ Thus, MOUD treatment using infection risk mitigation strategies, which include the provision of telehealth-based treatments,^29^ are indispensable for the foreseeable future.

It is also worth mentioning that after treatment intake onboard the TM-MTU, patients were offered the option of receiving TM MOUD at the CCHD brick-and-mortar clinic intermittently or continuously. This flexibility allowed for patient-driven determinations in treatment delivery, a factor of shared decision-making that may facilitate engagement and improve treatment outcomes.^30,31^ Anecdotally, several patients shared vignettes with their treatment team that signaled a strong preference for receiving treatment onboard the TM-MTU. Although this was not assessed objectively, a common theme was that they found TM-MTU treatment to be less stigmatizing and more compassionate than the care received from conventional (brick-and-mortar) treatment. Future studies will explore this dimension of patient acceptability of the TM-MTU clinical services.

Importantly, although no formal cost-effectiveness analyses were conducted, data support that the TM-MTU model of care is both sustainable and reproducible. Income generated by the service currently covers almost all the ongoing TM-MTU staffing and vehicle maintenance costs, which are supplemented by state opioid response grants.

Collectively, these data suggest that the implementation of mobile substance use treatment programs that use videoconferencing platforms have the potential to transform the delivery of MOUD in underserved rural areas by bringing the expertise of specialty trained addiction practitioners proximate to where patients live. This novel approach can be used to access marginalized and hard-to-reach populations and, ultimately, reduce overdose deaths in communities disproportionately affected by the opioid epidemic.

### Limitations

Despite its strengths, this study also has several limitations inherent to retrospective quality improvement analyses. Although there is heuristic utility in a comparison of TM-MTU treatment outcomes with those of other office-based TM MOUD programs, this study lacks the rigorous design of a well-controlled efficacy assessment. A comparative effectiveness trial was untenable at the inception of the program but will be conducted in future research to more rigorously assess the effectiveness of this novel intervention.

In addition, the nature of our collaboration with the CCHD and the urgency of the rural Maryland overdose epidemic drove our TM team’s decision to develop and implement this TM-MTU model very quickly. Thus, we did not collect rigorous implementation outcome measures a priori (eg, patient and staff acceptance and community adoption) that would be would be valuable in future work using implementation science frameworks.

Furthermore, this study was conducted in a rural area in the mid-Atlantic state of Maryland. Replication studies conducted as randomized clinical trials or by adopting more pragmatic real-world trial methods would validate the generalizability of the effectiveness of our model, an important next step in adopting this novel approach to standard MOUD care. It would also be important to study the model in larger states, where internet connectivity across larger distances could impact feasibility.

## Conclusions

A substantial effort has been made recently to improve access to lifesaving MOUD in urban and rural communities. To mitigate gaps in access to available treatment for OUD, we have tested a mobile service that travels to rural areas, equipped with on-site diagnostic and treatment services delivered via videoconferencing by physicians specialized in addiction medicine. Our data indicate that by combining the known effective approaches of TM and mobile treatment, our model is a viable and feasible approach to narrow the gaps in accessibility to TM MOUD in underserved rural areas. Finally, this clinical paradigm serves as a bellwether for MOUD treatment in a pandemic era that is commanding a focus on delivering safer, minimal-contact treatment.

## Notes

Supplement. **eTable 1.** Urine Toxicology Panel **eTable 2.** Demographic Characteristics for All Enrolled Patients

## References

[1] Faul M, Dailey MW, Sugerman DE, Sasser SM, Levy B, Paulozzi LJ. Disparity in naloxone administration by emergency medical service providers and the burden of drug overdose in US rural communities. Am J Public Health. 2015;105(3)(suppl):e26-e32. doi:10.2105/AJPH.2014.302520 25905856 PMC4455515

[2] Mack KA, Jones CM, Ballesteros MF. Illicit drug use, illicit drug use disorders, and drug overdose deaths in metropolitan and nonmetropolitan areas—United States. Am J Transplant. 2017;17(12):3241-3252. doi:10.1111/ajt.14555 29145698

[3] Mattick RP, Breen C, Kimber J, Davoli M, Breen R. Methadone maintenance therapy versus no opioid replacement therapy for opioid dependence. Cochrane Database Syst Rev. 2003;(2):CD002209. doi:10.1002/14651858.CD002209 12804430

[4] Mattick RP, Breen C, Kimber J, Davoli M. Buprenorphine maintenance versus placebo or methadone maintenance for opioid dependence. Cochrane Database Syst Rev. 2014;(2):CD002207. doi:10.1002/14651858.CD002207.pub4 24500948 PMC10617756

[5] Lister JJ, Weaver A, Ellis JD, Himle JA, Ledgerwood DM. A systematic review of rural-specific barriers to medication treatment for opioid use disorder in the United States. Am J Drug Alcohol Abuse. 2020;46(3):273-288. doi:10.1080/00952990.2019.1694536 31809217

[6] Rosenblatt RA, Andrilla CHA, Catlin M, Larson EH. Geographic and specialty distribution of US physicians trained to treat opioid use disorder. Ann Fam Med. 2015;13(1):23-26. doi:10.1370/afm.1735 25583888 PMC4291261

[7] Andrilla CHA, Coulthard C, Larson EH. Barriers rural physicians face prescribing buprenorphine for opioid use disorder. Ann Fam Med. 2017;15(4):359-362. doi:10.1370/afm.2099 28694273 PMC5505456

[8] Andrilla CHA, Moore TE, Patterson DG, Larson EH. Geographic distribution of providers with a DEA waiver to prescribe buprenorphine for the treatment of opioid use disorder: a 5-year update. J Rural Health. 2019;35(1):108-112. doi:10.1111/jrh.12307 29923637

[9] Langabeer JR, Stotts AL, Cortez A, Tortolero G, Champagne-Langabeer T. Geographic proximity to buprenorphine treatment providers in the U.S. Drug Alcohol Depend. 2020;213:108131. doi:10.1016/j.drugalcdep.2020.108131 32599495

[10] Uscher-Pines L, Huskamp HA, Mehrotra A. Treating patients with opioid use disorder in their homes: an emerging treatment model. JAMA. 2020;324(1):39-40. doi:10.1001/jama.2020.3940 32459292 PMC7905739

[11] Barash D. Telemedicine in substance use disorder treatment. Health Aff (Millwood). 2019;38(2):331-331. doi:10.1377/hlthaff.2018.05435 30715974

[12] Weintraub E, Greenblatt AD, Chang J, Himelhoch S, Welsh C. Expanding access to buprenorphine treatment in rural areas with the use of telemedicine. Am J Addict. 2018;27(8):612-617. doi:10.1111/ajad.12805 30265425

[13] Weintraub E, Greenblatt AD, Chang J, . Outcomes for patients receiving telemedicine-delivered medication-based treatment for opioid use disorder: a retrospective chart review. Heroin Addict Relat Clin Probl. 2021;23(2):5-12. 33551692 PMC7861202

[14] University of Maryland Shore Regional Health. Community health needs assessment and implementation plan. Published 2019. Accessed July 30, 2021. https://www.umms.org/shore/community/assessment-implementation-plan

[15] Hall G, Neighbors CJ, Iheoma J, . Mobile opioid agonist treatment and public funding expands treatment for disenfranchised opioid-dependent individuals. J Subst Abuse Treat. 2014;46(4):511-515. doi:10.1016/j.jsat.2013.11.002 24468235

[16] Krawczyk N, Buresh M, Gordon MS, Blue TR, Fingerhood MI, Agus D. Expanding low-threshold buprenorphine to justice-involved individuals through mobile treatment: addressing a critical care gap. J Subst Abuse Treat. 2019;103:1-8. doi:10.1016/j.jsat.2019.05.002 31229187 PMC6612429

[17] Ogrinc G, Davies L, Goodman D, Batalden P, Davidoff F, Stevens D. Squire 2.0 (Standards for Quality Improvement Reporting Excellence): revised publication guidelines from a detailed consensus process. Am J Crit Care. 2015;24(6):466-473. doi:10.4037/ajcc2015455 26523003

[18] Wysocki VH, Resing KA, Zhang Q, Cheng G. Mass spectrometry of peptides and proteins. Methods. 2005;35(3):211-222. doi:10.1016/j.ymeth.2004.08.013 15722218

[19] Harris PA, Taylor R, Thielke R, Payne J, Gonzalez N, Conde JG. Research electronic data capture (REDCap): a metadata-driven methodology and workflow process for providing translational research informatics support. J Biomed Inform. 2009;42(2):377-381. doi:10.1016/j.jbi.2008.08.010 18929686 PMC2700030

[20] Schiff DM, Nielsen T, Hoeppner BB, . Assessment of racial and ethnic disparities in the use of medication to treat opioid use disorder among pregnant women in Massachusetts. JAMA Netw Open. 2020;3(5):e205734. doi:10.1001/jamanetworkopen.2020.5734 32453384 PMC7251447

[21] Henzen A, Moeglin C, Giannakopoulos P, Sentissi O. Determinants of dropout in a community-based mental health crisis centre. BMC Psychiatry. 2016;16(1):111. doi:10.1186/s12888-016-0819-4 27095462 PMC4837516

[22] Stein MD, Cioe P, Friedmann PD. Buprenorphine retention in primary care. J Gen Intern Med. 2005;20(11):1038-1041. doi:10.1111/j.1525-1497.2005.0228.x 16307630 PMC1490248

[23] Darke S, Campbell G, Popple G. Retention, early dropout and treatment completion among therapeutic community admissions. Drug Alcohol Rev. 2012;31(1):64-71. doi:10.1111/j.1465-3362.2011.00298.x 21426420

[24] Monico LB, Mitchell SG, Gryczynski J, . Prior experience with non-prescribed buprenorphine: role in treatment entry and retention. J Subst Abuse Treat. 2015;57:57-62. doi:10.1016/j.jsat.2015.04.010 25980599 PMC4561018

[25] Varisco T, Shen C, Thornton D. Chronic prescription opioid use predicts stabilization on buprenorphine for the treatment of opioid use disorder. J Subst Abuse Treat. 2020;117:108073. doi:10.1016/j.jsat.2020.108073 32811630 PMC7451120

[26] Pizzicato LN, Hom JK, Sun M, Johnson CC, Viner KM. Adherence to buprenorphine: an analysis of prescription drug monitoring program data. Drug Alcohol Depend. 2020;216:108317. doi:10.1016/j.drugalcdep.2020.108317 33035714

[27] Fareed A, Vayalapalli S, Casarella J, Drexler K. Effect of buprenorphine dose on treatment outcome. J Addict Dis. 2012;31(1):8-18. doi:10.1080/10550887.2011.642758 22356665

[28] Bloom BR, Nowak GJ, Orenstein W. “When will we have a vaccine?” understanding questions and answers about Covid-19 vaccination. N Engl J Med. 2020;383(23):2202-2204. doi:10.1056/NEJMp2025331 32897660

[29] Alexander GC, Stoller KB, Haffajee RL, Saloner B. An epidemic in the midst of a pandemic: opioid use disorder and COVID-19. Ann Intern Med. 2020;173(1):57-58. doi:10.7326/M20-1141 32240283 PMC7138407

[30] Korthuis PT, McCarty D, Weimer M, . Primary care-based models for the treatment of opioid use disorder: a scoping review. Ann Intern Med. 2017;166(4):268-278. doi:10.7326/M16-2149 27919103 PMC5504692

[31] Stacey D, Légaré F, Lewis K, . Decision aids for people facing health treatment or screening decisions. Cochrane Database Syst Rev. 2017;4(4):CD001431. doi:10.1002/14651858.CD001431.pub5 28402085 PMC6478132

