# Supplementary material for: Mobile Telemedicine for Buprenorphine Treatment in Rural Populations With Opioid Use Disorder
Source: JAMA Netw Open. 2021 Aug 27;4(8):e2118487. doi: 10.1001/jamanetworkopen.2021.18487 (PMC8397932; doi:10.1001/jamanetworkopen.2021.18487)
Supplement: Supplement. — eTable 1. Urine Toxicology Panel eTable 2. Demographic Characteristics for All Enrolled Patients [file jamanetwopen-e2118487-s001.pdf]

Supplemental Online Content

Weintraub E, Seneviratne C, Anane J, et al. Mobile telemedicine for buprenorphine treatment in rural populations with opioid use disorder. *JAMA Netw Open*. 2021;4(8):e2118487. doi:10.1001/jamanetworkopen.2021.18487

eTable 1. Urine Toxicology Panel

eTable 2. Demographic Characteristics for All Enrolled Patients

This supplemental material has been provided by the authors to give readers additional information about their work.

© 2021 Weintraub E et al. *JAMA Network Open.*

eTable 1. Urine Toxicology Panel

Drug class based on chemical composition	Analyte
Alcohol	Alcohol SCR
	ETG Screen (Ethyl Glucuronide)
	Ethyl Sulfate
Amphetamine	Amphetamine SCR
	Methamphetamine
Barbiturates	Barbiturates SCR
	Benzodiazepines SCR
	Butalbital
Benzodiazepines	Benzodiazepines SCR
	7-Aminoclonazepam
	Lorazepam
	Oxazepam
	Nordiazepam
	Temazepam
	Alpha-OH-alprazolam
Non-benzodiazepine sedatives	Zolpidem Metabolite
Cannabinoids	Cannabinoids SCR
	Carboxy THC/ THC COOH
Cocaine	Benzoylecgonine/ Cocaine SCR
NMDA receptor antagonists	Phencyclidine SCR
	Ecstasy SCR
Opioids	6-Acetylmorphine SCR
	Methadone SCR
	EDDP SCR
	Opiates SCR

© 2021 Weintraub E et al. *JAMA Network Open.*

	Oxycodone SCR
	Buprenorphine SCR
	Norbuprenorphine
	Fentanyl
	Norfentanyl
	6-AM (Heroin Mtb)
	Codein
	Morphine
	Tramadol
	O-Desmethyltramadol
	Oxymorphone
	Noroxycodone
	Hydrocodone
	Norhydrocodone
	Hydromorphone

© 2021 Weintraub E et al. *JAMA Network Open.*

eTable 2. Demographic Characteristics for All Enrolled Patients

Characteristic	TM-MTU patients (N=94)	<50% TM-MTU visits (N=24)	All enrolled patients (N=118)	*P-value
Average Age in years (S.D.)	36.53 (9.78)	38.08 (11.74)	36.85 (10.17)	0.51
Gender (men; N, %)	59 (62.77)	14 (58.33)	73 (61.83)	0.65
Race (N, %):				
White	71 (75.53)	19 (75.53)	90 (76.27)	0.71
Black or African American	15 (15.96)	5 (20.83)	20 (16.95)	0.57
Multiracial	2 (2.13)	0	2 (1.69)	n/a
Other/Unknown	7 (6.38)	0	6 (5.08)	n/a
Ethnicity (N, %):				
Hispanic	3 (3.19)	1 (4.17)	4 (3.39)	0.82
Not Hispanic	90 (95.74)	19 (79.17)	109 (92.37)	0.01
Unknown	1 (1.06)	4 (16.67)	5 (4.24)	0.001
Patients with positive urine screens at baseline (N, %)				
Alcohol	14 (17.95)	3 (13.04)	17 (16.83)	0.59
Amphetamines	15 (19.23)	2 (8.7)	17 (16.83)	0.24
Barbiturates	0	0	0	n/a
Benzodiazepines	6 (7.69)	2 (8.70)	8 (7.92)	0.97
Nonbenzodiazepine sedative	0	1 (4.35)	1 (0.99)	0.07
Cannabinoids	37 (47.44)	7 (30.43)	44 (43.56)	0.15
Cocaine	10 (12.82)	3 (13.04)	13 (12.87)	0.98
NMDA receptor antagonists	1 (1.28)	0	1 (0.99)	n/a
Opioids (including (nor)buprenorphine)[1]	73 (93.59)	23 (100.00)	96 (95.05)	0.22

[1] Includes prescription and non-prescription

© 2021 Weintraub E et al. *JAMA Network Open.*

Opioids (excluding (nor)buprenorphine)	45 (57.69)	10 (43.48)	55 (53.92)	0.28
Buprenorphine dosing (in mg, S.D.)				
Average initial daily dose	11.09 (4.84)	11.24 (4.67)	11.12 (4.83)	0.90
Average daily dose across treatment	15.24 (5.24)	15.58 (5.26)	15.31 (31)	0.78
Maximum daily prescribed dose	36	36	36	n/a
Minimum daily prescribed dose	4	4	4	n/a

*P-value for the comparison between TM-MTU patients and patients who had <50% visits on TM-MTU

© 2021 Weintraub E et al. *JAMA Network Open.*
